# Impact of probiotics on sleep quality and mood states in patients with insomnia: a systematic review and meta-analysis

**DOI:** 10.3389/fmicb.2025.1596990

**Published:** 2025-07-16

**Authors:** Yi Liu, Yunfeng Yu, Shenghua Lu, Kang Tan, Pengfei Jiang, Pei Liu, Qinghua Peng

**Affiliations:** ^1^School of Traditional Chinese Medicine, Hunan University of Chinese Medicine, Changsha, China; ^2^The First Clinical College of Traditional Chinese Medicine, Hunan University of Chinese Medicine, Changsha, China; ^3^Quzhou Hospital Ophthalmology Center, Zhejiang Medical and Health University, Quzhou, China

**Keywords:** insomnia, sleep disorders, probiotics, sleep quality, mood states, systematic review, meta-analysis

## Abstract

**Background:**

Probiotics have garnered increasing attention for their potential role in managing insomnia. This meta-analysis evaluated the effects of probiotics on sleep quality and mood in patients with insomnia.

**Methods:**

Eight public databases were searched to identify relevant randomized controlled trials (RCTs) published before December 2024. Data from included studies were extracted, and their risk of bias was assessed. Meta-analysis, sensitivity analysis, and publication bias assessment were conducted using Review Manager 5.3 software. The certainty of evidence was evaluated using Grading of Recommendations Assessment, Development and Evaluation (GRADE) system.

**Results:**

Six studies, encompassing 424 patients, were included. Compared to control groups, the probiotic interventions were associated with a significant reduction in Pittsburgh Sleep Quality Index (PSQI) (mean difference [MD] −2.10, 95% confidence interval [CI] −3.86 to −0.34, *p* = 0.02, GRADE: moderate) and Hamilton Depression Scale (HAMD) (MD −7.72, 95% CI −14.55 to −0.89, *p* = 0.03, GRADE: very low) scores. However, no significant effects were observed on total sleep time (MD 43.70, 95% CI −18.07 to 105.46, *p* = 0.17, GRADE: very low), sleep efficiency (MD 0.41, 95% CI −1.67 to 2.48, *p* = 0.70, GRADE: moderate), or sleep latency (MD −4.74, 95% CI −9.42 to −0.05, *p* = 0.05, GRADE: moderate). Additionally, no significant differences in total adverse events were noted between probiotic and control groups. Funnel plots indicated no publication bias for PSQI, sleep efficiency, or sleep latency, whereas potential publication bias was detected for HAMD, total sleep time, and total adverse events.

**Conclusion:**

Probiotic interventions improved sleep quality and reduced depressive symptoms in patients with insomnia without increasing the risk of adverse events. These findings highlight the potential of probiotics as complementary treatments for insomnia. However, due to the limited sample size, further high-quality clinical studies are warranted to confirm these findings.

**Systematic review registration:**

https://www.crd.york.ac.uk/PROSPERO/view/CRD420251077696, identifier CRD420251077696.

## 1 Introduction

Insomnia, a prevalent sleep disorder, is characterized by difficulty initiating or maintaining sleep, or experiencing non-restorative sleep, occurring at least three nights per week for a minimum of 3 months, with associated daytime dysfunction (Buysse, 2013; De Simone et al., 2023). This condition encompasses both primary and secondary insomnia, distinguished by whether sleep disturbances arise independently or from other medical, psychiatric, or substance-related factors. Differentiating between primary and secondary insomnia is often challenging due to overlapping comorbidities (McCrae and Lichstein, 2001). Consequently, the International Classification of Sleep Disorders, Third Edition (ICSD-3), and the Diagnostic and Statistical Manual of Mental Disorders, Fifth Edition (DSM-5), classify chronic and frequent sleep continuity disturbances as insomnia, without distinguishing between primary and secondary types (Gauld et al., 2022; Perlis et al., 2022). Excessive arousal, widely regarded as the primary pathological mechanism of insomnia (De Simone et al., 2023), results in abnormal electroencephalogram frequencies, hindering the brain’s transition from wakefulness to sleep due to sustained high activity levels during rest (Riemann et al., 2015). Mechanisms such as increased autonomic nerve function activity, overactivity of the hypothalamic-pituitary-adrenal (HPA) axis, increased inflammatory factors release, and neurotransmitter imbalances disrupt sleep regulation, contributing to insomnia (Matenchuk et al., 2020; Cryan and Dinan, 2012). Insomnia is widespread among the general population, with approximately 30–50% of adults experience insomnia at some point, as reported in recent studies (Aernout et al., 2021). Chronic insomnia can lead to fatigue, impaired concentration, and an increased risk of diabetes and cardiovascular disease, and may even contribute to sudden death or suicide (The Lancet Diabetes Endocrinology, 2024). Notably, insomnia serves as an early and significant indicator of suicidal behavior (Lu et al., 2024). A study of 5,692 American adults revealed that individuals with sleep disturbances were 5.1 times more likely to contemplate suicide, 9.1 times more likely to plan suicide, and 7.5 times more likely to attempt suicide compared to those without sleep issues (Wojnar et al., 2009). Current treatments for insomnia primarily include cognitive-behavioral therapy and pharmacological interventions (Chan et al., 2021; Qaseem et al., 2016). Although hypnotic medications effectively improve sleep, prolonged use may result in dependence or abuse (de Zambotti et al., 2018). Many patients prefer non-pharmacological approaches (Vincent and Lionberg, 2001). While cognitive-behavioral therapy is an established non-pharmacological treatment, its high cost limits accessibility (Palmqvist et al., 2007). Therefore, safe and effective adjunctive treatment strategies for insomnia are needed.

In recent years, the relationship between gut microbiota and insomnia has gained considerable attention. Insomnia can impair intestinal function and barrier integrity. Previous studies have reported decreased levels of serum markers of intestinal integrity—such as diamine oxidase (DAO), d-lactic acid (D-LA), intestinal fatty acid-binding protein (I-FABP), and endothelin (ET)—in patients with insomnia, suggesting a potential barrier damage (Cai et al., 2024). Conversely, gut microbiota and their metabolites are involved in regulating sleep. For instance, gut microbiota can produce hormones and neurotransmitters from dietary components such as polysaccharides, polyphenols, and peptides, which interact with neurons to promote or inhibit sleep (Yang et al., 2023). These metabolites influence brain function via the bloodstream and vagal afferent pathways (Godos et al., 2020). Circadian rhythm disturbances are also closely linked to gut microbiota dysregulation and insomnia (Matenchuk et al., 2020). The composition and function of gut microbiota exhibit circadian rhythm fluctuations. Disruptions in circadian rhythms can alter the secretion of neurotransmitters and hormones by interfering with the intestinal flora and stimulating intestinal activity, including melatonin and serotonin, thereby exacerbating insomnia symptoms (Tian Y. et al., 2022; Thaiss et al., 2014). Additionally, chronic insomnia further desynchronizes the biological clock from the external environment, worsening circadian rhythm disorders and gut microbiota dysfunction (Voigt et al., 2016). These findings highlight the bidirectional relationship between insomnia and gut microbiota disruption.

Given the role of gut microbiota in sleep regulation, probiotics have emerged as a potential treatment for insomnia (Frazier and Chang, 2020). Several clinical studies have reported favorable effects of probiotics on sleep and explored their underlying mechanisms. A study involving 230 participants showed that Lactobacillus plantarum JYLP-326 alleviated insomnia and depression in college students, potentially by regulating gut microbiota metabolites (Zhu et al., 2023). A randomized, double-blind controlled trial reported that 4 weeks of prebiotic yeast mannan supplementation significantly improved gut health and sleep quality in healthy adults (Tanihiro et al., 2023). This effect may result from modulation of gut-derived propionic acid and GABA, alongside stimulation of neurotransmitters such as 5-hydroxytryptamine (5-HT), which influence sleep quality (Tanihiro et al., 2023). Another double-blind, randomized controlled trial with 38 healthy young participants found that a multi-strain probiotic mixture reduced Pittsburgh Sleep Quality Index (PSQI) over time (Marotta et al., 2019). Considering that anxiety and depression are significant risk factors for insomnia (Shan et al., 2024), the effects of probiotics on emotional states in patients with insomnia warrant attention. Daily supplementation with *Lactobacillus plantarum* PS128 has been shown to improve sleep quality and reduce anxiety and depression in patients with insomnia (Ho et al., 2021). Thus, the regulation of negative emotions by probiotics may represent a mechanism for improving sleep.

These findings suggest that probiotics enhance human sleep quality and mood states by modulating gut microbiota metabolites and neurotransmitters. However, given the limited number of high-quality, large-scale clinical trials, a comprehensive evaluation of the existing evidence is warranted. Therefore, this meta-analysis was conducted to assess the effects of probiotics on sleep quality and mood states in patients with insomnia and to provide a scientific foundation for their potential application as adjunctive therapies.

## 2 Materials and methods

This study adhered to the Preferred Reporting Items for Systematic Reviews and Meta-Analyses (PRISMA) guidelines (Radua, 2021), and was registered in PROSPERO (CRD420251077696).

### 2.1 Literature search

A comprehensive search was conducted across eight databases: Embase, PubMed, Cochrane Library, Web of Science, China National Knowledge Infrastructure (CNKI), Wanfang, China Science and Technology Journal Database (VIP), and Sinomed. Subject terms included “insomnia” and “probiotics,” supplemented by terms from the Medical Subject Headings (MeSH) database. The search was restricted to (Title/Abstract) fields, using the following formula: [(Probiotic OR Probiotics OR *Bacillus* OR *Bacillus Bifida* OR *Bifidobacteria* OR *Bifidobacterium* OR *Candida Robusta* OR *Clostridium Butyricum* OR *Enterococcu* OR *Lactobacill* OR *Lactobacillus Acidophilus* OR *Lactobacillus Amylovorus* OR Lactic Acid Bacteria OR Natto Bacteria OR *Saccharomyces Capensis* OR *Saccharomyces Cerevisiae* OR *Saccharomyces Italicus* OR *Saccharomyces Oviformis* OR *Saccharomyces Uvarum* Var Melibiosus OR *S. Cerevisiae* OR *S Cerevisiae* OR *Streptococcus Thermophiles* OR Yeast) AND (Insomnia OR Sleeplessness OR Sleep)]. The search spanned from database inception to December 31, 2024, with no language or other restrictions. Additionally, to ensure a comprehensive literature retrieval and minimize the risk of missing relevant studies, we manually screened the reference lists of all included studies and relevant reviews.

### 2.2 Inclusion and exclusion criteria

Inclusion criteria were as follows: (1) Participants: patients diagnosed with insomnia per the ICSD-3 or DSM-5. (2) Intervention and comparison: Control groups received no treatment or conventional treatment only, while experimental groups received probiotic treatment in addition to control group interventions. (3) Outcomes: The primary efficacy outcome was the PSQI score. Secondary efficacy outcomes included sleep latency, total sleep time, sleep efficiency, and the Hamilton Depression Scale (HAMD). Safety outcomes were assessed based on total adverse events. (4) Study design: Randomized controlled trials (RCTs).

Exclusion criteria were as follows: (1) Duplicate publications, in cases of multiple reports using the same data, only the most complete version was included. (2) Abstract-only publications without full text. (3) Studies with insufficient or ambiguous data.

### 2.3 Literature screening

Two researchers (YL and YY) independently screened the literature using NoteExpress 3.0 (Beijing Aegean Sea Joy Technology Co., Ltd.) according to the inclusion and exclusion criteria. Duplicate publications, studies irrelevant to the research topic, and those lacking complete data were sequentially excluded to determine the final set of included studies. Disagreements during screening or selection were resolved by a third researcher (SL).

### 2.4 Data extraction

Two researchers (YL and YY) independently extracted basic information (author names, sample sources, sample size, male proportion, mean age, disease duration, intervention, and treatment duration) and outcome data from each study. A third researcher (SL) cross-checked the extracted data to ensure accuracy.

### 2.5 Risk of bias assessment

The risk of bias in included studies was independently evaluated by two researchers (YL and YY) using the Cochrane Risk of Bias Tool in Review Manager 5.3. Assessment covered random sequence generation, allocation concealment, blinding, incomplete outcome data, selective reporting, and other biases, with each item classified as low, unclear, or high risk. A third researcher (SL) reviewed the evaluations to ensure consistency.

### 2.6 Statistical analysis

Meta-analysis was performed using Review Manager 5.3 software. Continuous variables were analyzed using the mean difference (MD) and 95% confidence interval (CI) as effect measures. Dichotomous variables were analyzed using the relative risk (RR) and 95% CI. A fixed-effects model was applied when heterogeneity was low (*I*^2^ < 50%), and a random-effects model was used when heterogeneity was high (*I*^2^ > 50%). Statistical significance was set at *P* < 0.05. Subgroup analyses of outcomes with significant heterogeneity were conducted based on clinical factors, including participant source, proportion of male patients, age, disease course, intervention, and treatment duration, to identify heterogeneity sources. A leave-one-out sensitivity analysis was performed to further investigate the individual sources of heterogeneity and assess the results robustness. Results were considered robust if the combined effect size remained stable after sequentially excluding each study. Publication bias was evaluated using funnel plots, with symmetrical scatter point distribution indicating no bias. The certainty of evidence for each outcome was assessed using Grading of Recommendations Assessment, Development and Evaluation (GRADE) system, considering risk of bias, inconsistency, indirectness, imprecision, and publication bias. Evidence was graded as high, moderate, low, or very low based on these domains, with downgrading for limitations (e.g., high risk of bias, heterogeneity, or imprecision) and upgrading for factors increasing confidence. This approach ensured transparent and consistent evidence grading.

## 3 Results

### 3.1 Literature selection

**FIGURE 1:**
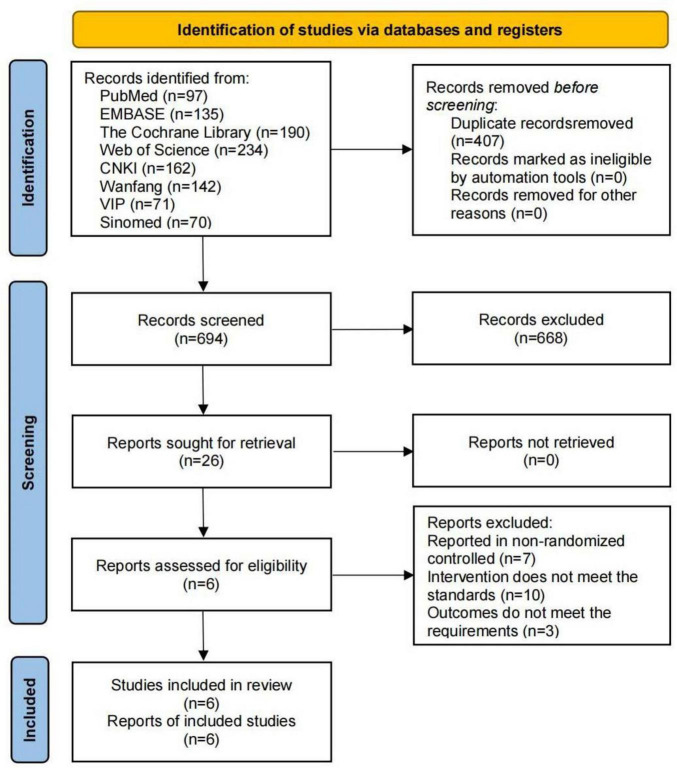
Flowchart of literature screening process.

A total of 1,101 relevant studies were identified through the database search: 97 from PubMed, 135 from Embase, 190 from the Cochrane Library, 234 from Web of Science, 162 from CNKI, 142 from Wanfang, 71 from VIP, and 70 from Sinomed. During screening, 407 studies were excluded due to duplication, and 688 were excluded for not meeting the inclusion criteria. Six studies were ultimately included (Ding et al., 2024; Yan et al., 2024; Yu and Zhan, 2023; Byun et al., 2018; Murakami et al., 2024; West et al., 2020), as shown in Figure 1.

### 3.2 Basic characteristics of included studies

Six clinical studies, published between 2018 and 2024, were included: three from China, one from Japan, one from South Korea, and one from Australia. These studies enrolled 424 patients with insomnia, with a mean proportion of male patients of 29.5% and a mean age of 39.3 years. Of these, 223 patients received probiotic interventions, while 201 received no probiotic treatment, as detailed in Table 1.

**TABLE 1:** Basic characteristics of included studies.

Study	Source of participants	Sample size (E/C)	Male ratio (%)	Mean age (years)	Disease duration	Intervention	Comparison	Treatment duration (days)
Byun et al. (2018)	Korea	30/10	25.0	48.7	/	*Lactobacillus sakei* B2-16	Placebo	28
Ding et al. (2024)	China	53/55	0.0	27.8	18.3 d	*Saccharomyces boulardii*	None	8
Murakami et al. (2024)	Japan	61/65	49.2	46.4	/	*Bifidobacterium adolescentis* SBT2786	Placebo	28
West et al. (2020)	Australia	29/29	25.9	41.7	/	*Lactobacillus acidophilus* DDS-1	Placebo	14
Yan et al. (2024)	China	20/12	37.5	40.6	5.85 y	Multi-strain probiotics	Placebo	28
Yu and Zhan (2023)	China	30/30	43.3	35.6	/	Bifidobacterium active bacteria capsules	None	35

E, experimental group; C, control group. Baseline data, such as sex, age, and disease duration between the experimental and control groups were comparable for each included study.

### 3.3 Risk of bias assessment

**FIGURE 2:**
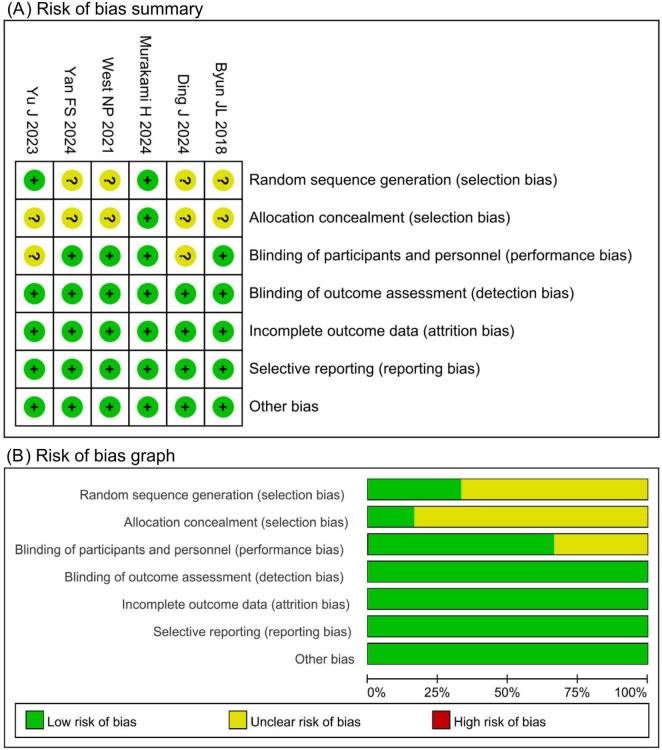
Risk of bias assessment. **(A)** Risk of bias summary. **(B)** Risk of bias graph.

The Cochrane Risk of Bias tool was used to evaluate the included studies. Four studies had an unclear risk of bias in random sequence generation due to insufficient reporting of randomization methods. Five studies had an unclear risk of bias in allocation concealment due to unreported methods. Two studies had an unclear risk of bias in participant blinding owing to the absence of a placebo design. All other domains were assessed as low risk. Of the six included studies, four were judged to have an overall low risk of bias, whereas two had an overall high risk of bias, as shown in Figure 2.

### 3.4 Meta-analysis

#### 3.4.1 Primary efficacy outcomes

**FIGURE 3:**
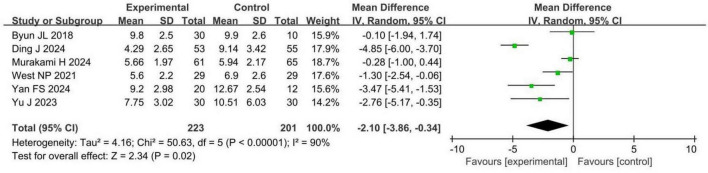
Forest plots of meta-analysis for PSQI. PSQI, Pittsburgh sleep quality index.

Six RCTs involving 424 patients with insomnia compared PSQI scores between probiotic and control groups. Four studies were assessed as having a low risk of bias, while two had a high risk. PSQI scores were significantly reduced in the probiotic group compared to the control group (MD −2.10, 95% CI −3.86 to −0.34, *p* = 0.02, *I*^2^ = 90%), as shown in Figure 3.

A 2.1-point reduction in PSQI scores is clinically significant, indicating improved sleep quality and reduced sleep disturbances, which can enhance patients’ daily functioning, wellbeing, and quality of life. These findings suggest that probiotic interventions may benefit sleep quality in patients with insomnia, with both statistical and clinical relevance.

#### 3.4.2 Secondary efficacy outcomes

**FIGURE 4:**
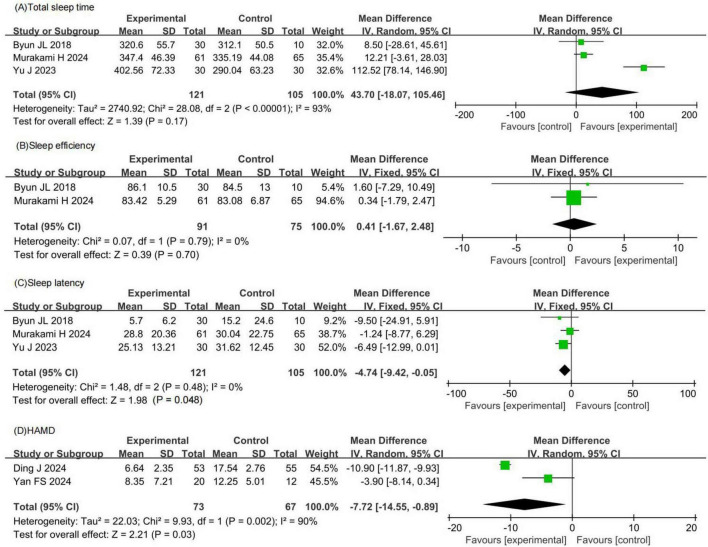
Forest plots of meta-analysis for secondary efficacy outcomes. **(A)** Total sleep time. **(B)** Sleep efficiency. **(C)** Sleep latency. **(D)** HAMD. HAMD, Hamilton depression scale.

Three RCTs involving 226 patients with insomnia compared total sleep time between probiotic and control groups. Two studies had a low risk of bias, while one had a high risk. Total sleep time in the probiotic group was comparable to that in the control group (MD 43.70, 95% CI −18.07 to 105.46, *p* = 0.17, *I*^2^ = 93%), as shown in Figure 4A.

Two RCTs involving 166 patients with insomnia compared sleep efficiency between probiotic and control groups. Both studies had a low risk of bias. Sleep efficiency in the probiotic group was comparable to that in the control group (MD 0.41, 95% CI −1.67 to 2.48, *p* = 0.70, *I*^2^ = 0%), as shown in Figure 4B.

Three RCTs involving 226 patients with insomnia compared sleep latency between probiotic and control groups. Two studies had a low risk of bias, while one had a high risk. Sleep latency in the probiotic group was comparable to that in the control group (MD −4.74, 95% CI −9.42 to −0.05, *p* = 0.05, *I*^2^ = 0%), as shown in Figure 4C.

Two RCTs involving 140 patients with insomnia compared HAMD scores between probiotic and control groups. One study had a low risk of bias, while one had a high risk. HAMD scores in the probiotic group were significantly lower than those in the control group (MD −7.72, 95% CI −14.55 to −0.89, *p* = 0.03, *I*^2^ = 90%), as shown in Figure 4D.

Based on the meta-analysis results, the observed effect sizes for total sleep time, sleep efficiency, and sleep latency were not statistically significant and showed high heterogeneity, indicating that probiotics did not produce clear or consistent improvements in these sleep parameters. However, this meta-analysis demonstrated that probiotics significantly reduced HAMD scores by 7.72 points. This decrease is clinically meaningful as it indicates a significant improvement in depressive symptoms, which are often comorbid with insomnia.

#### 3.4.3 Safety outcomes

**FIGURE 5:**
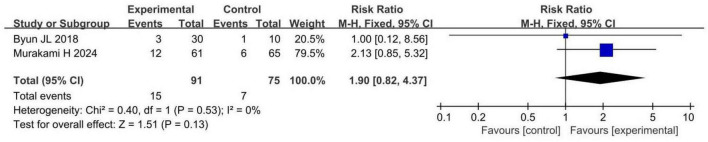
Forest plots of meta-analysis for total adverse events.

Two RCTs involving 166 patients with insomnia compared total adverse events between probiotic and control groups. Both studies had a low risk of bias. Total adverse events in the probiotic group were comparable to those in the control group (RR 1.90, 95% CI 0.82 to 4.37, *p* = 0.13, *I*^2^ = 0%), as shown in Figure 5.

### 3.5 Heterogeneity analysis

#### 3.5.1 Subgroup analysis

Subgroup analyses were conducted to explore heterogeneity in PSQI scores, examining the impact of participant source and treatment duration. Studies were grouped by participant source (China, Japan, South Korea, and Australia). Probiotic interventions significantly reduced PSQI scores in participants from China (MD −4.24, 95% CI −5.16 to −3.33, *p* < 0.00001, *I*^2^ = 36%) and Australia (MD −1.30, 95% CI −2.54 to −0.06, *p* = 0.04, *I*^2^ = 0%), but not in those from Japan (MD −0.28, 95% CI −1.00 to 0.44, *p* = 0.45, *I*^2^ = 0%) or South Korea (MD −0.10, 95% CI −1.94 to 1.74, *p* = 0.92, *I*^2^ = 0%). The reduced heterogeneity within each subgroup suggests that that differences in PSQI outcomes may be related to the participant’s geographic source.

Studies were also grouped by treatment duration (≤ 14 days or ≥ 28 days). Probiotic supplementation for either duration did not significantly affect PSQI scores. The lack of significant findings may be attributed to limited sample size and high heterogeneity. Although neither treatment duration subgroup showed statistically significant differences in PSQI scores, both demonstrated a trend toward PSQI reduction, with *p*-values approaching the threshold for significance, as shown in Table 2.

**TABLE 2:** Subgroup analyses of the primary efficacy outcome—PSQI.

Subject	Subgroup	Number of studies	I^2^	MD (95%CI)	*p-*value
Source of participants	China	3	36	−4.24 (−5.16, −3.33)	<0.00001
	Japan	1	0	−0.28 (−1.00, 0.44)	0.45
	Korea	1	0	−0.10 (−1.94, 1.74)	0.92
	Australia	1	0	−1.30 (−2.54, −0.06)	0.04
Treatment duration	≤ 12 days	2	94	−3.08 (−6.56, 0.40)	0.08
	≥ 28 days	4	76	−1.48 (−3.12, 0.17)	0.08

#### 3.5.2 Sensitivity analysis

A leave-one-out sensitivity analysis was performed for PSQI, total sleep time, and sleep latency to explore heterogeneity and assess result robustness. No single study significantly contributed to PSQI heterogeneity, confirming robust results. For total sleep time, heterogeneity was attributed to a study by Yu and Zhan (2023), which had a high risk of bias due to methodological issues (Yu and Zhan, 2023). After excluding this study, total sleep time remained comparable between the two groups (MD 11.64, 95% CI −2.92 to 26.19, *p* = 0.12, I^2^ = 0%), indicating robust results. Sleep latency showed no significant heterogeneity, and the sensitivity analysis confirmed its robustness. Sensitivity analysis was not feasible for sleep efficiency, HAMD scores, or total adverse events due to the inclusion of only two studies.

In summary, PSQI heterogeneity was associated with participant source, while total sleep time heterogeneity stemmed from a study with a high risk of bias. Heterogeneity sources for sleep efficiency, HAMD scores, and total adverse events could not be identified due to limited studies.

### 3.6 Publication bias

**FIGURE 6:**
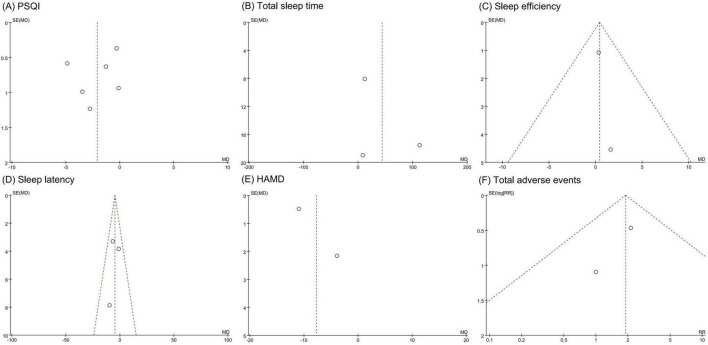
Funnel plots of publication bias assessment. **(A)** PSQI. **(B)** Total sleep time. **(C)** Sleep efficiency. **(D)** Sleep latency. **(E)** HAMD. **(F)** total adverse events. PSQI, Pittsburgh sleep quality index; HAMD, Hamilton depression scale.

Funnel plots for PSQI, sleep efficiency, and sleep latency showed symmetrical scatter distributions, indicating no publication bias. However, funnel plots for total sleep time, HAMD score, and total adverse events displayed asymmetrical distributions, suggesting potential publication bias, as shown in Figure 6.

### 3.7 Certainty of evidence

The GRADE system indicated moderate certainty for PSQI, sleep efficiency, and sleep latency, low certainty for total adverse events, and very low certainty for total sleep time and HAMD scores. However, the overall recommendation strength was strong, as detailed in Table 3.

**TABLE 3:** Certainty of evidence.

Outcome	Risk of bias	Inconsistency	Indirectness	Imprecision	Others	MD/RR (95% CI)	Certainty of evidence
PSQI	None	Serious^a^	None	None	None	2.10 (−3.86, −0.34)	Moderate
Total sleep time	None	Serious^a^	None	Serious^b^	Serious^c^	43.70 (−18.07, 105.46)	Very Low
Sleep efficiency	None	None	None	Serious^b^	None	0.41 (−1.67, 2.48)	Moderate
Sleep latency	None	None	None	Serious^b^	None	−4.74 (−9.42, −0.05)	Moderate
HAMD	None	Serious^a^	None	Serious^b^	Serious^c^	−7.72 (−14.55, −0.89)	Very Low
Total adverse events	None	None	None	Serious^b^	Serious^c^	1.90 (0.82, 4.37)	Low

*^a^*Highly heterogeneous;

*^b^*insufficient sample size;

*^c^*publication bias. PSQI, Pittsburgh sleep quality index; HAMD, Hamilton depression scale; MD, mean difference; CI, confidence interval.

## 4 Discussion

### 4.1 Background and significance

Insomnia is a widespread condition that significantly impacts human health, particularly among older adults (Milner et al., 2021). Chronic insomnia can impair physical function and mental wellbeing and is associated with severe consequences, including increased risks of suicide and sudden death (Aernout et al., 2021; Roth, 2007). Although the therapeutic potential of probiotics for insomnia has attracted growing interest, high-quality evidence supporting their benefits remains scarce. To the best of our knowledge, this is the first systematic review and meta-analysis evaluating the efficacy of probiotics in the treatment of insomnia. Our findings revealed that the probiotic interventions were associated with a significant reduction in PSQI and HAMD scores compared to controls. However, no significant improvements were observed on total sleep time, sleep efficiency, or sleep latency. Furthermore, the analysis indicated no significant difference in adverse event rates between the probiotic and control groups, suggesting that probiotic interventions are generally safe.

### 4.2 Impact of probiotics on sleep quality

The PSQI, a widely used measure of sleep quality, served as the primary efficacy outcome. Our results demonstrated that probiotics significantly reduced the PSQI score by 2.1 points compared to the placebo or no treatment, suggesting that probiotics effectively improved sleep conditions. Sensitivity analysis confirmed the robustness of these results. Subgroup analysis by participant source revealed significant PSQI reductions in participants from China (MD −4.24, 95% CI −5.16 to −3.33, *p* < 0.00001, *I*^2^ = 36%) and Australia (MD −1.30, 95% CI −2.54 to −0.06, *p* = 0.04, *I*^2^ = 0%), but not in those from Japan (MD −0.28, 95% CI −1.00 to 0.44, *p* = 0.45, *I*^2^ = 0%) or South Korea (MD −0.10, 95% CI −1.94 to 1.74, *p* = 0.92, *I*^2^ = 0%). These findings indicate that participant source contributed to PSQI heterogeneity. Variations in insomnia risk factors across racial and regional groups, such as higher risks among African Americans and Caucasians, may explain these differences (Kim et al., 2012; Roth et al., 2011). Thus, the effects of probiotics on sleep may differ across populations in different countries and regions. Furthermore, this study found no significant effects of probiotics on secondary sleep indicators, including total sleep time, sleep efficiency, and sleep latency. However, these non-significant results may be due to the limited sample sizes. Therefore, the impact of probiotics on these additional sleep metrics should be further investigated in future studies.

The mechanism by which probiotics may help treat insomnia likely involve the regulation of neurotransmitters, hormones, and inflammatory responses. From a neurophysiological perspective, the vagus nerve serves as a direct communication pathway between the gut and the brain. Microbial metabolites, such as short-chain fatty acids (SCFAs) can stimulate the vagus nerve and influence neural activity in sleep-related brain regions, including the hypothalamus. For example, Lactobacilli have been shown to enhance GABA synthesis, thereby inhibiting central nervous system excitability and promoting deeper sleep (Li et al., 2024). Fermented milk enriched with *Lactobacillus brevis* DL1-11 has been reported to elevate GABA levels and improve sleep duration and latency in mice (Yu et al., 2020).

Hormonal regulation also plays a role. Gut microbiota influence the synthesis of serotonin (5-HT) and melatonin—key hormones in sleep regulation. Spore-forming *Clostridium* species can convert tryptophan into serotonin precursors, indirectly promoting melatonin secretion and circadian rhythm regulation (Gao et al., 2018; Wang et al., 2023). In one study, *B. coagulans* BC99 improved sleep quality by increasing brain 5-HT levels (Sun et al., 2025). Furthermore, modulation of the HPA axis has been implicated (Levenson et al., 2015). For instance, *Bifidobacterium breve* CCFM1025 improves sleep quality by regulating serum daidzein levels, affecting HPA axis activity (Lan et al., 2023).

The anti-inflammatory effects of probiotics may further contribute to improvements in sleep quality. Previous studies have shown that disruptions in gut microbiota can trigger inflammation, leading to elevated levels of pro-inflammatory factors such as interleukin (IL)-6 and tumor necrosis factor (TNF-α). This inflammation may impair sleep by activating the microbiota-gut-brain axis (MGBA) (Zheng et al., 2023). Conversely, probiotics can inhibit intestinal inflammation by modulating the balance of gut microbiota and restoring the integrity of the intestinal mucosal barrier (Bermudez-Brito et al., 2012; Cristofori et al., 2021; Ng et al., 2009). For example, the probiotic formulation SLAB51 has been shown to restore gut-brain axis hormone levels and alleviate insomnia-induced inflammation in both peripheral tissues and the brain (Sánchez et al., 2017). Additionally, supplementation with GABA-producing probiotics has been found to reduce the levels of pro-inflammatory cytokines IL-8 and TNF-α (Zhao et al., 2022). In summary, probiotics may improve sleep quality by regulating neurotransmitters, hormones, and inflammatory factors.

### 4.3 Impact of probiotics on mood state

Negative emotional states, including depression and anxiety, are not only risk factors for insomnia but may also be exacerbated by it. Data from a Chinese survey revealed a strong associations between insomnia and both depression (OR 11.29, 95% CI 9.58–13.29) and anxiety (OR 10.98, 95% CI 8.78–13.72) (Shan et al., 2024). A longitudinal cohort study further confirmed that insomnia predicts the persistence of anxiety and depression (Meaklim et al., 2023). These findings suggest that negative emotions such as depression and anxiety may create a vicious cycle with insomnia, resulting in progressive deterioration in sleep quality. While the primary focus of this study was the effect of probiotics on sleep quality, their potential influence on negative emotional states—particularly depression and anxiety—was also evaluated. Notably, previous meta-analyses have demonstrated the effectiveness of probiotics in regulating mood disorders. One meta-analysis of 23 randomized controlled trials found that probiotics significantly alleviated symptoms of depression (SMD −0.96, 95% CI −1.31 to −0.61) and anxiety (SMD −0.59, 95% CI −0.98 to −0.19) (Asad et al., 2024). Therefore, our study does not explore the broader effects of probiotics on depression and anxiety in the general population. Instead it focuses specifically on their impact on negative emotions in individuals with insomnia. As the included studies reported only HAMD scores and did not provide anxiety-related data, this meta-analysis assessed the effects of probiotics on depression alone.

Our meta-analysis revealed that, compared to placebo or no treatment, probiotics significantly reduced the HAMD score by 7.72 points. This finding highlights the potential of probiotics in lowering the risk of depression and provides additional insights into their role in improving sleep quality. Previous studies support these findings. Two randomized controlled trials reported that healthy individuals who consumed probiotics (*Lactobacillus casei* strain Shirota and *Lactobacillus gasseri* CP2305) were able to maintain satisfactory sleep quality even under depression or anxiety (Takada et al., 2017; Nishida et al., 2017). Another study demonstrated that *Bifidobacterium breve* CCFM1025 enhanced subjective sleep quality in patients with insomnia and reduced sleep disturbances, accompanied by a more pronounced reduction in stress markers (Lan et al., 2023). Collectively, these results suggest that probiotics exert meaningful effects in alleviating negative emotions such as anxiety and depression, aligning with the broader goal of treating insomnia.

The mechanisms by which probiotics alleviate depression may involve the inhibition of inflammatory responses, the reduction of cortisol levels, and the enhancement of brain-derived neurotrophic factor (BDNF) expression. First, probiotics downregulate pro-inflammatory factors such as TNF-α, IL-1β, and IL-6, thereby reducing microglial overactivation and inflammatory damage in the hippocampus, ultimately improving depressive symptoms (Park et al., 2018; Tian P. et al., 2022). Second, probiotics lower cortisol levels via neuroendocrine pathways, thus reducing the incidence of mood disorder-related symptoms (Tian P. et al., 2022; Qian et al., 2024). Third, probiotics can promote nerve regeneration by increasing BDNF levels, contributing to the alleviation of depressive symptoms (Suda and Matsuda, 2022). These findings elucidate the biological pathways through which probiotics may reduce depression risk and further support their therapeutic potential in the management of insomnia.

### 4.4 Safety analysis

Adverse events occurred in 16.5% (15/91) of patients in the probiotic group and 9.3% (7/75) in the control group, with no significant difference between groups. Among the included studies, Byun et al. (2018) reported four adverse events: three in the probiotic group (abdominal discomfort, headache, drowsiness) and one in the control group (drowsiness). Drowsiness was attributed to sleep deprivation, not probiotics or placebo. The abdominal discomfort and headache were mild, suggesting good tolerability. Murakami et al. (2024) conducted a comprehensive safety assessment of probiotics through medical interviews, physical measurements (height and weight), physiological tests (blood pressure and pulse rate), and relevant examinations (hematology, blood biochemistry, and urinalysis). They found no significant differences in the incidence of adverse events between the probiotic and placebo groups. Moreover, changes in clinical test parameters remained within physiological ranges in both groups (Murakami et al., 2024). Additionally, a long-term study involving 156 participants found no significant abnormalities in blood cell counts, biochemical tests, urinalysis, or electrocardiograms (Lee et al., 2021). Although adverse events occurred in 2.6% (2/78) of cases, they were mild, self-limiting, and did not lead to treatment discontinuation (Lee et al., 2021). Collectively, these findings support the conclusion that probiotics are safe and well-tolerated for the treatment of insomnia.

### 4.5 Limitations and perspectives

This study provides biologically plausible explanations and clinical evidence supporting the use of probiotics to improve sleep and mood in individuals with insomnia. However, several limitations must be acknowledged. First, the inclusion of only six studies with 424 participants may have reduced result precision and generalizability. Second, variability in probiotic strains, dosages, and intervention durations introduced clinical heterogeneity, but the limited number of studies precluded subgroup analysis or meta-regression to explore these factors. Consequently, optimal strains and dosages remain unclear. Third, five studies lacked reported concealment methods, and two omitted blinding procedures, increasing risks of selection and performance biases. The mean participant age of 39.3 years limits applicability to older adults, who exhibit distinct physiological and insomnia-related characteristics.

Future research should address these limitations. Larger, well-designed randomized controlled trials with diverse populations are required to enhance precision and generalizability. Rigorous reporting of concealment and blinding methods will minimize bias. Systematic investigation of probiotic strains, dosages, and intervention durations is essential to identify optimal regimens. Including older adults will clarify the efficacy and safety of probiotics in this demographic. These efforts will support the development of personalized probiotic therapies for insomnia.

## 5 Conclusion

Probiotic interventions improved sleep quality and reduced depressive symptoms in patients with insomnia without increasing the risk of adverse events. These findings highlight the potential of probiotics as complementary treatments for insomnia. However, the small sample size necessitates further evaluation through large-scale, low-bias clinical trials to confirm the efficacy and optimal application of probiotics.

## Data Availability

The original contributions presented in this study are included in this article/supplementary material, further inquiries can be directed to the corresponding authors.
